# A multi-stage emergency supplies pre-allocation approach for freeway black spots: A Chinese case study

**DOI:** 10.1371/journal.pone.0240372

**Published:** 2020-10-08

**Authors:** Siliang Luan, Qingfang Yang, Zhongtai Jiang, Wei Wang, Chao Chen

**Affiliations:** 1 School of Transportation, Jilin University, Changchun, P. R. China; 2 Jilin Research Center for Intelligent Transportation System, Changchun, P. R. China; 3 School of Automotive Engineering, Dalian University of Technology, Dalian, P. R. China; Univerza v Mariboru, SLOVENIA

## Abstract

This study presents a multi-stage random regret minimization (RRM) model as an emergency rescue decision support system to determine the emergency resource pre-allocation schedule for the freeway network. The proposed methodology consists of three steps: (1) improved accident frequency approach to identify the black spots on the freeway network, (2) stochastic programming (SP) model to determine the initial allocation plan sets, and (3) regret-based model in the logarithmical specification to select the most minimal regret one considering the factors of the response time, total cost and demand. The model is applied to the case study of 2014–2016 freeway network in Shandong, China. The results show that the random regret minimization (RRM) model can improve the full-compensation of SP model to a certain degree. RRM in logarithmical specification performs lightly better than random utility maximization (RUM) and RRM in the linear-additive specification in this case. This approach emerges as a valuable tool to help decision makers to allocate resources before traffic accident occurs, with the aim of minimizing the total regret of their decisions.

## 1. Introduction

Since the subject of emergency management emerged, it has already become a worldwide-noticeable theme for natural or man-made disasters. Most of the emergency planning models are designed to respond to irregular and unpredictable events, but ignore that road incidents have already become the most major cause of death [1]. Every year more than 1.25 million people are dead and approximate 50 million suffer injuries because of road safety accidents [2]. The critical issues in traffic accidents are how to provide immediate assistance to the victims as soon as possible and how to mitigate the effects of the incidents [3]. Emergency supply pre-allocation as the first step of the rescue impacts the quality of the relief efforts. There is also a great need for reliable decision-support models to help evaluate and improve the performance of such systems [4]. In this paper, a new way to set up pre-arranged emergency resources is established for decision makers.

Previous studies have developed several supply allocation models to support the decision-making process, but there is still an understanding on how to improve the pre-allocation process in emergency management. The design of our emergency resource pre-arrangement strategy includes the identification of black spots and the preparedness of rescue supplies in advance. Once the traffic incidents have occurred, the affected people’s assistance can be launched in an orderly manner.

In this paper, we take advantage of the historical accident records that can be inferred from the accident-prone areas. Moreover, summarizing the previous literature, we attempt to formulate models that are based on stochastic programming (SP) model. SP-based model is an appropriate tool for planning in the preparedness phase and has been successful in many applications. Notwithstanding the obvious success of the SP model for emergency management purposes, there is much scope for the characteristics of full compensation. It assumes that one of the attributes outperforms other attributes, which can compensate other attributes with bad performance greatly. Therefore, we introduce the random regret minimization (RRM) model to improve the defects of SP model. RRM’s contribution to decision support system (DSS) lies in its semi-compensatory characteristics based on regret theory (RT), which can avoid offsetting an equally large decline in the performance of another attribute.

The proposed methodology is unique and has three contributions as follows:

1. The improved accident frequency method associated with freeway road network helps to identify more risky areas. Given that the black spots on the freeway are known exactly, the accident probability is obtained more easily according to the prediction, and the limited supplies can be transferred to the areas where are prone to occur accidents in time. Methodologically, adding the method of accident identification to the spectrum of emergency rescue makes sense.
2. A new SP-RRM model for emergency resource pre-arrangement introduces the regret theory into emergency management, which provides a different perspective to look at emergency system decisions. Regret-based model intents to avoid the negative payoff and choose the final alternative that has already taken into account the foregone alternatives, which is adapted to emergency fields. Especially, the characteristics of semi-compensatory improve the SP-only approaches.
3. The studies example compares SP-RRM2010(in the logarithmic specification) with the SP-only, SP- Random Utility Maximization (RUM) and SP-RRM2008(in the linear-additive specification) for our discussed issue. The objective to be optimized is represented in terms of response time, allocation cost and demand.

The remainder of the paper is organized as follows. In Section 2, the relevant literature is reviewed. Section 3 illustrates the multi-stage emergency supplies pre-allocation approach. In Section 4, a Chinese case study for the application of our strategy is introduced. Section 5 presents conclusions and directions for further research.

## 2. Literature review

Our literature review consists of two sub-sections. The first sub-section reviews the recent studies that are emergency management related to resource allocation. The second sub-section reviews past researches that are tightly connected to the theory and methodology used in this paper.

### 2.1 Emergency resource allocation

Much of the effort in emergency supplies allocation focuses on natural disaster management. Haghani and Oh presented a formulation to solve a large-scale multi-commodity, multi-modal network problem with time windows in disaster relief management [5]. Fiedrich et al. minimized the total facilities after strong earthquakes and used the detailed descriptions of the operational areas and of the available resources to calculate the model for different disasters [6]. Sherali et al. considered comprehensively the aftermath of a natural disaster, terrorist attack, and so on, and stated a tight linear programming relaxation [7]. Dodo et al. put forward a linear program to minimize the overall risk of earthquakes and to avoid losses in the future earthquake [8]. Minciardi et al. presented two main phases to manage natural hazards: a pre-event phase is to reallocate resources closer to sites; the during event-phase is to manage the available resources in real-time. This approach is useful for the risk management of natural hazards [9]. Mete and Zabinsky proposed a stochastic optimization to store and distribute medical supplies in preparation for disasters [10]. In the paper by Rawls and Turnquist, a two-stage stochastic mixed integer program was presented to provide response strategies for hurricanes or other natural disasters, which was solved by Lagrangian L-shaped method (LLSM) [11]. In the paper by Wex et al., a decision support model in natural disaster management that minimizes the sum of completion time of incidents was developed and was solved by a heuristics algorithm that was selected among Monte Carlo-based heuristic, the joint application of 8 construction heuristics and 5 improvement heuristics [12].

Increased attention in the literature has been focused on allocating resources by Stochastic Program (SP) model. Primary concerns are often the development of the allocation of medical resources such as ambulances to the emergency occurrence areas [13–16]. Baker et al. proposed an integer, non-linear mathematical programming model to allocate emergency medical service ambulances to meet the government-mandated response-time criterion and also reflect the criterion for budget and workload [17]. In addition, Ahmed formulated a 0–1 integer linear programming problem to allocate resources to maximize the total maintenance effectiveness for highway maintenance management [18]. Yin presented a min-max bi-level programming model to allocate tow trucks among patrol beats to contribute to the traffic incident management systems on the freeway [19]. In 2008, Yin extended his original model and proposed a mixed-integer nonlinear programming model to minimize the expected loss of different incidents [20]. Ozbay et al. introduced the concept of quality of service during a potential incident and put forward an SP model with probabilistic constraints to respond the incidents and allocate resources [21]. In the paper by Garrido et al. [22], a spatio-temporal stochastic process model for the logistics of a flood emergency can help the decision makers well to deliver enough supplies to satisfy the demands before and after a flood occurs. Feng et al. [23] optimized the hospital emergency departments(Eds) system in Taiwan and used multi-objective stochastic mathematical model for the limited medical resources allocation. This mathematical model was solved by an integrated non-dominated sorting genetic algorithm (NSGA) II.

### 2.2 Regret theory and regret-based model

In contributing to the continued discussion on emergency management in transportation research, and following this generally accepted methodological approach, in this paper, we introduce the notion of regret theory and apply RRM model instead of only SP-based model in DSS. It is widely assumed that regret is based on a kind of emotion that individuals do not satisfy their expectation, that is, the chosen alternative performs worse than the non-chosen one. Hence, Bell utilized this choice behavior to develop the regret theory to provide assistance to make a good decision with regard to tradeoff and lotteries in order to avoid regret in 1982 [24]. In search of discrete choice models, the concept of regret has recently attracted the attention of several scholars in transportation research [25–40]. Regret-based models assume that individuals minimize regret rather than maximize utility when they face several choice alternatives.

Chorus et al. introduced RRM model into transportation research [25]. The original regret-based model is in a linear function of attribute differences between the considered alternative and the best of foregone alternatives. In 2010, Chorus proposed a regret-based discrete choice-model in logarithmic specification instead of the discontinuous function [26]. This new model not only considered all foregone alternative, but also can use standard discrete-choice software packages. Later, G-RRM model replaced the fixed constant of one in the attribute level regret function of the RRM2010 model by regret-weight variable [33]. Cranenburgh et al. developed the classical RRM model and allowed a shape parameter μ to be estimated [35].

This fundamental work has led to an upsurge of applications of regret-based models in different fields. In term of the transportation research, it contains departure times [41], travel mode [42], route choices [28, 36, 43] and freight transport [32]. These literatures performed in this present study indicates that these applications focus on the individual choice from the user’s point of view and contribute to the user’s service system. However, few of the applications are in the context of emergency resource allocation. This issue belongs to the decision support system, and the decision makers are policymakers that have the duty to select the optimal determinate alternative to help people who may suffer the accidents.

A total of fifteen literature in Table 1 summarizes their choice type, data type, and model. Two papers do not use statistical methods, only analyzing the specification methodologically.

**Table 1:** Summary of feature used in selected papers.

Lead Author (Year)	Choice type	Data type	Model
Kaplan & Prato (2012) [29]	Travel route	RP	RRM2010
Chorus (2012a) [27]			RUM, RRM2008
Chorus (2012b) [28]	Travel route	SP	RRM2010
Chorus et al. (2013) [30]	Travel route	SP	RUM, RRM2010, contextual concavity model
Hensher et al. (2013) [31]	Travel mode	SP	RUM, RRM2010
Chorus (2014) [33]	Travel route	SP	G-RRM (logarithmic)
Boeri et al. (2014) [32]	Transportation mode, time, cost, punctuality of the transport	SP	RUM, RRM2010
Hess et al. (2014) [34]	Information acquisition	SP	RUM, RRM
An et al. (2015) [42]	Travel mode	SP	Hybrid model
Cranenburgh et al. (2015) [35]	Shopping location	SP	μRRM (logarithm), PRRM (logarithm)
Rasouli & Harry (2017) [37]	Parking fee	SP	RRMax, RRSum, RRlog
Li & Huang (2017) [36]	Travel route	SP	RUM, RRM2010
Chorus and Cranenburgh (2018) [38]	Ten datasets	SP	RUM, RRM2008, RRM2010, G-RRM, μRRM
Rasouli & Harry (2018a) [39]			RRM2008, RRM2010
Rasouli & Harry (2018b) [40]	Shopping destination, travel mode	SP	Regret-rejoice model

## 3. Mathematical formulation

One of the topics in the development of emergency management concerns the analysis of traffic accident data. A fundamental property of the traffic accident data is its enormousness and complexity, which is hard to infer accident-prone areas. To start with, introducing the improved accident frequency method gives the operators the way to identify the black spots on the freeway. Then we attempt to address an SP-RRM model to allocate emergency supplies before the accidents occur. In our model, multiple potential scenarios with various demand for supplies are allowed, and the response time, the total cost and the demand are assumed to be the major factors to make decisions.

### 3.1 The identification of black spots on the freeway

The identification of black spots contributes to the safety and the quality of service of the road network. The results of road accident statistics stress the need for more systematic mechanisms for accident analysis and prediction. The main goal of the identification of black spots is not only to find out the high-risk areas for potential accidents, but also can guarantee the next work concerned about resource arrangement.

Inherently, freeway networks constitute complex dynamic systems impacted by various uncertain factors. Black spot is not just “a point”, it can represent a point, a road section or an area [44]. In this paper, we stipulate that the black spot is a certain road section and put forward the improved accident frequency method to identify the black spots on the freeway. Given the critical number of accidents *CN* as the identification criterion, the number of accidents in a certain section is larger than the critical value, so it is considered as the prone accident occurrence location.

We define λ as the average number of accidents in unit sections.

$$\lambda =\frac{\sum m_{i}}{n}$$ (1)

Where *m*_*i*_ is the number of accidents on the road section *i* and *n* is the total number of the unit road section.

We define the critical number of accidents as *CN*, which is regarded as the accident threshold for the black spots. Assume the Eq (2) obey Poisson distribution and *u* is the confidence level. If the confidence level *u*_(1−*α*)/2_ is 95%, the critical number of accidents *CN* is:

$$CN=\lambda +u_{(1-\alpha )/2}\cdot \sqrt{\lambda },i=1,2,\ldots ,n$$ (2)

If the actual number of traffic accidents per year is larger than the critical number of accidents *CN*, that road section seems to be the black spot. However, this road segmentation method results in considerable errors as it may divide one whole accident-prone into two sections. Hence, the original fixed segmentation method needs to be modified by the cut-sectional technology to improve the defect of missing some black spots. The principle of this non-fixed length accident representation of black spots is to move some unit sections with a great number of accidents to a suitable area or integrate into one area on the basis of the distribution of their locations, in case of ignoring the potential black spots.

### 3.2 A SP-RRM model for pre-allocating emergency supplies at the black spots

Usually, the government decision-makers and scholars studying for emergency management prefer to use stochastic programming (SP) model for emergency resource allocation. SP model is a linear-additive specification with the maximum or minimum objective function. SP-only based models may potentially capture a valid choice mechanism in some decision contexts because SP model has the characteristics of full-compensation, that is, the attributes with better performance compensate for the other attributes with worse performance. For that very reason, many scholars tried to use different weights or penal coefficients to improve the significance of some factors. Indeed, this is an effective way to some extent, but we might doubt the process of calibration of the parameters. The parameters are obtained by referring to existing literatures or relying on personal experiences. If experts as the decision-makers express their preference based on their knowledge and empirical studies, the estimated results of the weight of considerable factors are likely to be more convincing. Our specific model not only provides a new way of thinking to make related emergency decisions, but improves the process of the calibration of the parameters.

Regret-based models have been introduced in several fields such as route choices, travel information acquisition choices, parking lot choices, shopping location choices and so on. Most researches related to regret-based models have in common that the decision makers are individuals with different personal attributes. Decision makers prefer to choose a regretless plan rather than a maximum utility schedule in emergency management.

Original RRM model is proposed by Chorus in 2008 [25] and it is called RRM2008 in the next contexts. The main idea of the RRM model is the compassion of different attributes of every alternative and to select the alternative with the minimal regret value. This method comprehensively considers every attribute’s performance and balance the overall situations. It can be expressed as:

$$\varphi_{x}(x_{i},x_{j})=\max\left\{0,\beta_{x}\cdot (x_{j}-x_{i})\right\}$$ (3)

$$\varphi_{y}(y_{i},y_{j})=\max\left\{0,\beta_{y}\cdot (y_{j}-y_{i})\right\}$$ (4)

$$\varphi_{z}(z_{i},z_{j})=\max\left\{0,\beta_{z}\cdot (z_{j}-z_{i})\right\}$$ (5)

Where *φ* is the linear regret function for altribute *x*,*y*,*z* for alternative *i*,*j*. Let *β* represent the estimated parameter of alternative *x*,*y*,*z*. And the regret associated with alternative *i* when compared to *j* is equal to:

$$R_{ij}=\varphi_{x}(x_{i},x_{j})+\varphi_{y}(y_{i},y_{j})+\varphi_{z}(z_{i},z_{j})$$ (6)

The regret of alternative *i*,*j*,*k* is:

$$R_{i}=\max\left\{R_{ij},R_{ik}\right\}$$ (7)

$$R_{j}=\max\left\{R_{ji},R_{jk}\right\}$$ (8)

$$R_{k}=\max\left\{R_{ki},R_{kj}\right\}$$ (9)

Finally, we select the minimal regret among *R*_*i*_,*R*_*j*_,*R*_*k*_.

In 2010, Chorus illustrated that the previous regret-based model (RRM2010 for short) has two limitations [26]: first, the previous model is anticipated with respect to only the best of foregone alternatives, but the new model assumes that regret is experienced about each foregone alternative that performs well. Second, the logarithmic specification’s likelihood function is smooth, unlike the non-smooth characteristics of previous model. The new model’s estimation doesn’t rely on handwritten code anymore. Therefore, he formulated a new model specification, and it is:

$$RR_{i}=R_{i}+\varepsilon_{i}=\sum_{j\neq i}\sum_{m}\ln[1+\exp(\beta_{m}(x_{jm}-x_{im}))]+\varepsilon_{i}$$ (10)

To map regret into choice probabilities, both model specifications assumed that regret is a stochastic variable. Its error terms are independently Gumbel distributed, and the probability of the choice models is:

$$P_{i}=\frac{\exp(-R_{i})}{\sum_{j=1\ldots J}\exp(-R_{j})}$$ (11)

Where *i*,*j* are alternatives and *i*,*j*∈*J*. *P*_*i*_ is the selected probability of the alternative *i*.

The fitting surface of logarithmic function is shown in S1 Fig. ln[1+exp(*β*_*m*_(*x*_*jm*_−*x*_*im*_))] is a monotonically increasing function of *β*_*m*_ and (*x*_*jm*_−*x*_*im*_). The regret value at the attribute level depends on the importance of the attributes and the difference between the two alternatives on the comparison of the properties. The estimated parameter *β*_*m*_ signifies the importance of the attribute *m*. When the chosen alternative *i* outperforms unselected alternative *j*, i.e., (*x*_*jm*_−*x*_*im*_)<0, the regret value is closer to 0. On the contrary, when the performance of the selected alternative *i* is not as good as the unselected scheme *j*, the larger the value of (*x*_*jm*_−*x*_*im*_) is, the greater the regret value is.

In line with these findings, we decide to use the RRM model in logarithmical specification for the emergency rescue discipline. All alternative plans are obtained from the SP model with the constraint of the probability of traffic accidents on the freeway network. RRM model is applied to get the optimum resources arrangement strategy, in response to different bad weather scenarios.

#### 3.2.1 Assumptions

We make the following assumptions about this model:

1. This model only considers resource allocation on hypothetical traffic accidents under different weather. Natural disasters such as earthquakes, hurricanes, floods, etc. are not considered.
2. We assume that the supply locations are connected to the demand locations, their connected roads are not damaged and the travel time can be predicted.
3. In this model, the demand location of the freeway network is the accident black spot (the location at which the high probability of accident occurs). The black spot is simplified to the particle point of the road segment.

#### 3.2.2 Notations of parameters and variables

To formulate our model before, we need to establish the model’s parameters and variables in Table 2.

**Table 2:** Notification.

**Indices and index sets**	
*I*	Set of all the supply locations *i*∈*I*
*J*	Set of all the black spots *j*∈*J*
**parameters**	
*p*_*j*_(*ξ*)	Accident probability of black spots *j* under the scenario *ξ*
*t*_0*ij*_	Emergency resources transportation time from supply location *i* to black spot *j*
${\bar{t}}_{i}$	The average processing time after receiving the alarm at the supply location *i*
*a*_*i*_	The maximum stock capacity of the supply location *i*
*A*	Number of total available emergency supplies
*B*	The maximum budget for the inventory and procurement in the system.
*r*	The minimal number of emergency resources that can handle a minor accident independently
*d*_*j*_(*ξ*)	Demand for emergency supplies in location *j* under the scenario *ξ*
*G*_*i*_	Service level of supply locations *i*. Grade 1 is the top of them, i.e., the supply location has the best rescue service. Grade 3 is the bottom of them, i.e., the supply location has the worst rescue ability. $G_{i}=\{\begin{array}{c} 3\;\mathrm{Grade}\;1\\ 2\;\mathrm{Grade}\;2\\ 1\;\mathrm{Grade}\;3 \end{array}$
*C*_*s*_	The inventory cost of emergency supplies
*C*_*b*_	The procurement cost of emergency supplies
*C*	The attribute of total cost, includes inventory and procurement costs
*T*	The attribute of response time. The response time includes reaction time after receiving the alarm and travel time from zone *i* to zone *j*.
*D*	The attribute of demand for emergency supplies
*β*	The estimated parameter of the attributes.
*l*_*ij*_	The shortest distance from zone *i* to zone *j*.
*v*(*ξ*)	The average transportation velocity under the scenario *ξ*.
*t*_*ij*_(*ξ*)	Transportation time from zone *i* to zone *j* under the scenario *ξ*.
*δ*_*ij*_(*ξ*)	Congestion delay index from zone *i* to zone *j* under the scenario *ξ*.
**Variables**	
*θ*_*ij*_(*ξ*)	1, if black spot *j* is covered by supply location *i*; 0, otherwise.
*x*_*ij*_(*ξ*)	The number of emergency supplies, sent to location *j*, originated from a supplier in location *i* under the scenario *ξ*.
*ε*	An independent and identically distributed (i.i.d.) error term.
*RR*(*ξ*)	The total regret value under the scenario *ξ*.
*R*_*T*_(*ξ*)	The regret of the attribute *T* under the scenario *ξ*.
*R*_*C*_(*ξ*)	The regret of the attribute *C* under the scenario *ξ*.
*R*_*D*_(*ξ*)	The regret of the attribute *D* under the scenario *ξ*.

We introduced the concept of scenario *ξ* during the modeling, which illustrates the future possible conditions [45]. Since the distinct characteristics of incidents cause different demands for resources and travel time, it is necessary to discuss emergency response schemes according to the specific emergency type. The scenario-based applications have grown extensively into the uncertainty emergency issues such as the railway infrastructure maintenance [46], the flood emergency logistics preparation [47] and highway transportation industry [48]. In our paper, the approach is based on the Bayesian Networks. Since the approach of Bayesian Networks has been discussed in detail in a number of previous papers, we don’t present deeply how to classify the scenarios [49].

As different bad weather usually leads to traffic congestion, we introduce this congestion delay index that is used widely to evaluate the current traffic situation in China, and we apply this index to modify the velocity of rescue vehicles. In China, different cities have their definition and standard for the congestion delay index. In order to avoid ambiguity, applying the definition of congestion delay index proposed by Autonavi company as in our model then gives the following calculation form:

$$\mathrm{Congestion}\;\mathrm{delay}\;\mathrm{index}=\frac{\mathrm{Peak}\;\mathrm{travel}\;\mathrm{time}}{\mathrm{Free}\;\mathrm{flow}(\mathrm{non}‐\mathrm{congestion})\;\mathrm{travel}\;\mathrm{time}}$$

This parameter is obtained by analyzing historical records and current floating cars feedback, and makes some sense to modify the travel time from supply location *i* to demand point *j*. The formulation of transportation time is:

$$t_{ij}(\xi )=\delta_{ij}(\xi )\cdot \frac{l_{ij}}{v(\xi )}$$ (12)

#### 3.2.3 The optimization formulation

With the parameters and variables previously defined, we now present SP model to obtain initial plans.

$$\min\sum_{i\in I}\sum_{j\in J}\theta_{ij}(\xi )p_{j}(\xi )t_{ij}(\xi )x_{ij}(\xi )$$ (13)

Subject to

$$\sum_{j\in J}x_{ij}(\xi )\leq a_{i},\forall i\in I$$ (14)

$$\sum_{j\in J}x_{ij}(\xi )\geq r,\forall i\in I$$ (15)

$$\sum_{i\in I}x_{ij}(\xi )\leq d_{j},\forall j\in J$$ (16)

$$\theta_{i}=\{\begin{array}{cc} 0 & t_{ij}(\xi )>t_{0ij}-\bar{t_{i}}\\ 1 & t_{ij}(\xi )\leq t_{0ij}-\bar{t_{i}} \end{array},\forall i\in I$$ (17)

$$x_{ij}(\xi )\geq 0,\;\mathrm{and}\;\mathrm{integer}\;\forall i\in I$$ (18)

The objective function of this stage (13) incorporates the total response time and accident probability of black spots in order to provide limited emergency supplies to the demand locations. The goal of the objective function is to minimize the response time and the number of resources. Constraint (14) ensures that the storage of emergency supplies in the supply location cannot exceed the capacity. Constraint (15) ensures that the number of emergency supplies in every supply location can handle an accident independently. Constraint (16) guarantees that the emergency supplies that may be distributed to zone *j* cannot exceed the actual demand. Constraint (17) states that if the black spot *j* is within the coverage of supply location *i*, *θ*_*ij*_ = 1; otherwise, *θ*_*ij*_ = 0. Constraint (18) illustrates the non-negative nature and the integer requirement of *x*_*ij*_(*ξ*).

Recall that the regret-based models contribute to the development of the decision support system. We propose the RRM model in the lograthimic specification to select the optimum alternative.

minRR(ξ) (19)

$$RR(\xi )=R_{T}(\xi )+R_{C}(\xi )+R_{D}(\xi )+\varepsilon$$ (20)

$$R_{T}(\xi )=\sum_{others\neq choice}\ln(1+\exp(\beta_{T}(T_{others}-T_{choice})))$$ (21)

$$R_{C}(\xi )=\sum_{others\neq choice}\ln(1+\exp(\beta_{C}(C_{others}-C_{choice})))$$ (22)

$$R_{D}(\xi )=\sum_{others\neq choice}\ln(1+\exp(\beta_{D}(D_{others}-D_{choice})))$$ (23)

$$T=\sum_{i\in I}\sum_{j\in J}t_{ij}(\xi )x_{ij}(\xi )$$ (24)

$$C=\sum_{i\in I}\sum_{j\in J}\left(C_{s}+C_{b}\right)G_{i}x_{ij}(\xi )$$ (25)

$$D=\sum_{j\in J}\left(d_{j}-x_{ij}(\xi \right))$$ (26)

Subject to:

$$\sum_{j\in J}x_{ij}(\xi )\leq a_{i},\forall i\in I$$ (27)

$$\sum_{i\in I}\sum_{j\in J}x_{ij}(\xi )\leq A$$ (28)

$$\sum_{j\in J}x_{ij}(\xi )\geq r,\forall i\in I$$ (29)

$$\sum_{i\in I}x_{ij}(\xi )\leq d_{j},\forall j\in J$$ (30)

$$\sum_{i\in I}\sum_{j\in J}\left(C_{s}+C_{b}\right)G_{i}x_{ij}(\xi )\leq B$$ (31)

$$x_{ij}(\xi )\geq 0,\;\mathrm{and}\;\mathrm{integer}\;\forall i\in I$$ (32)

The objective function (19) of the final stage is to select the allocation plan with the minimal regret value and formula (20) is the linear function to calculate the total regret value of an alternative plan. Formula (21)–(23) respectively presents the regret value of the attribute of response time, cost and demand under the scenario ξ by comparing every alternative. Eq (24) indicates the meaning of the attribute of response time. Eq (25) states that the total costs include the storage cost and acquisition cost of resources. Eq (26) defines that the attribute of demand equals the difference between actual demands and actual emergency supplies. If the actual dispatches don’t meet the need for demands at black spots, the regret value will increase. The limitations on the capacities of warehouses and the maximum number of emergency resources are represented by (27) and (28), respectively. Constraint (29) ensures that the number of emergency supplies in every supply location can handle an accident independently. Constraint (30) guarantees that the emergency supplies that may be distributed to zone *j* cannot exceed the actual demand. The maximum number of budgets *B* are taken into account by constraint (31). Constraint (32) illustrates the non-negative nature and the integer requirement of *x*_*ij*_(*ξ*).

Since *R*_*T*_(*ξ*),*R*_*C*_(*ξ*),*R*_*D*_(*ξ*) have different dimensions, it is necessary to use the unified dimension. We carry out non-dimensionalization of (*T*_*others*_−*T*_*choice*_), (*C*_*others*_−*C*_*choice*_) and (*W*_*others*_−*W*_*choice*_). This process is also called formatting the parameters. We use standardization method to convert them to unified dimension data. Standardized formula is as follows:

$$y_{i}=\frac{x_{i}-\bar{x}}{s}$$ (33)

Where $\bar{x}=\frac{1}{n}\sum_{i=1}^{n}x_{i}$ and $s=\sqrt{\frac{1}{n-1}\sum_{i=1}^{n}{\left(x_{i}-\bar{x}\right)}^{2}}$.

## 4. A case study

In this section, we illustrate the newly developed methodological insights and present an example to demonstrate the validity of our SP-RRM model. The data we used contains the three-year (2014–2016) traffic incidents on a rainy day in three Chinese cities, i.e., Linyi, Weifang and Rizhao where they occupied the highest traffic accident rate in Shandong province. These data are analyzed once before [50].

### 4.1 The identification of black spots on the freeway

We analyze a recent three-year accident portion of Shandong province and find that Linyi has the largest number of accidents. As Rizhao and Weifang are near Linyi according to the geographic location, we take these three cities as our study areas to identify the black spots. Linyi has four freeways, i.e., G2, G20, G22, and G25; Weifang embodies G18, G25 and G20; Rizhao includes G15, G1511 and G25. We take G2 as an example to present the process of the identification of black spots on the freeway. G2 has a length of 1261.99 kilometers from K540+195 to K709+800 and is across six provinces in all. In this paper, we only discuss G2 in the Linyi section.

#### 4.1.1 The critical value of accidents

We determine 1km as a unit length and divide G2 in the Linyi section into several units. And there are 170 road sections in total. The total number of accidents for every unit road section is 864.

The average number of accidents λ on a unit is:

$$\lambda =\frac{\sum m_{i}}{n}=\frac{864}{170}\approx 5$$

If the confidence level is 95%, the critical number of accidents *CN* is:

$$CN=\lambda +u_{(1-\alpha )/2}\cdot \sqrt{\lambda }=\lambda +1.96\sqrt{\lambda }\approx 10$$

#### 4.1.2 Initial identification process of black spots

By comparing the actual accidents and the critical value, we identified 27 black spots initially in Table 3.

**Table 3:** The initial result of the identification of black spots.

Original Pile No	Final Pile No	The amount of accident	Original Pile No	Final Pile No	The amount of accident
K542	K543	12	K581	K582	10
K545	K546	11	K599	K600	10
K546	K547	11	K600	K601	10
K547	K548	10	K604	K605	11
K551	K552	16	K609	K610	10
K553	K554	11	K613	K614	10
K555	K556	11	K615	K616	10
K556	K557	10	K616	K617	10
K563	K564	10	K617	K618	11
K564	K565	11	K639	K640	10
K567	K568	10	K642	K643	11
K577	K578	10	K649	K650	10
K578	K579	10	K681	K682	10
K580	K581	13	K707	K708	10

#### 4.1.3 The modification of the initial approach

Although most of the black spots have been identified initially, the rough results may ignore some hidden black spots owing to the fixed segmentation method. Thus, it is valuable to use cut section technology to modify the initial results. The approach mainly modifies two conditions in S2 Fig. The first condition is regarding the black spots that have been identified in the previous step. As can be seen, the K707-K708 is regarded as a black spot, for the number of accidents exceeds the critical number of accidents *CN*. However, the road sections around K707-K708 also have accidents and the total number of the accident is high. Hence, in light of the accident distribution of the adjacent road sections, we integrated them into a new road section and stated point A as the black spot for this new road section K704-K709. In terms of condition 2, the number of accidents of road section K570-K571 and K571-K572 is less than the critical number of accidents *CN* and they do not seem as the black spots. However, it is unreasonable to ignore the potential black spot, since the total number of accident between K570 and K572 is larger than the critical number of accidents *CN*. Therefore, K570-K572 is modified by the cut section technology as a new black spot.

Based on the modified approach, the final results are obtained as in Table 4.

**Table 4:** The final result of modification.

Black spots *j*		The number of accidents
Original pile No	Final pile No	
K542	K543	12
K545	K548	32
K551	K557	48
K561	K568	39
K570	K572	16
K575	K585	20
K588	K591	60
K599	K605	41
K609	K618	51
K622	K627	15
K639	K643	25
K647	K650	18
K681	K682	10
K704	K709	19

We identified 14 black spots on the G2 finally. The rest of freeway also applied the same way to identify the black spots.

#### 4.1.4 The calculation of accident probability of black spots

After considering the actual situation of every freeway, i.e., the traffic flow and the total number of accidents on every freeway, we calculate the probability of accident at black spot that is just a relative value. The method is expressed as follows.

$$p_{i}=w_{tf}\cdot w_{a}\cdot \frac{n_{i}}{\sum_{i}n_{i}}$$ (34)

Where *p*_*i*_ is the accident probability of black spot *i* and *n*_*i*_ is the number of accident of black spot *i*. *w*_*tf*_ denotes the weight of traffic flow, and *w*_*a*_ is the weight of accident and expresses by $w_{a}=\frac{m_{j}}{\sum_{j}m_{j}}$, where *m*_*j*_ is the total number of accidents of highway *j*.

In S3 Fig, the proportion of accidents on every freeway is counted. The final results of accident probability of black spots are shown in Table 5.

**Table 5:** The results of accident probability.

NO	Name	Original Pile No	Final Pile No	Accident Probability
1	G15	K704	K707	0.012234
2		K712	K715	0.015293
3		K742	K744	0.006117
4	G18	K363	K364	0.00534
⋮		⋮	⋮	⋮
10		K443	K445	0.00534
11	G2	K542	K543	0.006662
⋮		⋮	⋮	⋮
24		K704	K709	0.011104
25	G20	K101	K102	0.010531
⋮		⋮	⋮	⋮
36		K213	K216	0.038614
37	G22	K101	K103	0.005706
⋮		⋮	⋮	⋮
43		K159	K160	0.005706
44	G25	K1406	K1408	0.005464
⋮		⋮	⋮	⋮
61		K1604	K1606	0.010927
62	G1511	K16	K18	0.009786
⋮		⋮	⋮	⋮
72		K156	K158	0.009786

### 4.2 SP-RRM model for emergency supplies pre-allocation

#### 4.2.1 SP model for initial alternative plans

Usually, different types of supplies need to be pre-positioned in storage warehouses for different scenarios. In the example application in Section 4, we only consider one type of commodity(wreckers) on rainy days, but in general, the list of commodities might be several kinds of supplies. This case study focusing on preparedness for traffic accident threats in three cities serves to illustrate the SP model. To highlight the validity of our methodology, the case study is small enough to also be solved by a computer software package but detailed enough to be of interest as an illustration.

The natural scenario is considered, that is, the rainfall intensity is less than 2.5 *mm*⋅*h*^−1^. The average speed is 82.4 *km*/*h* and the average congestion index is 1.62. In the given case, 40 wreckers are available and 18 warehouses (road administration brigades and road administration squadrons) can store wreckers. The grade of road administration brigades is 1 and the grade of road administration squadrons is 2. The emergency manual in Shandong province stipulates that the maximum rescue time is 30 minutes and the average process time is 5 minutes. According to the time constraint, the major element *θ*_*ij*_ can judge whether the supply location *i* covers the black spot *j*. Genetic algorithm is one of the widely useful heuristic algorithms and it usually solves the SP model. By changing the number of iterations, initial scales of the population, etc., of the genetic algorithm, the alternative plans are obtained in Table 6. The values in Table 6 illustrate the number of rescue vehicles for black spots when this area occurs in the same condition. We coded our models in MATLAB2014a and obtained 9 plans as our alternatives in the second stage.

**Table 6:** The initial alternative plans by SP model.

Scheme No Black spot	1	2	3	4	5	6	7	8	9
**1**	0	1	1	1	0	0	2	0	0
**2**	0	0	2	2	0	0	1	0	1
**3**	0	0	0	0	1	2	0	0	0
**4**	0	0	0	0	0	0	0	0	0
**⋮**	⋮	⋮	⋮	⋮	⋮	⋮	⋮	⋮	⋮
**65**	1	1	2	0	0	0	0	0	1
**66**	1	2	1	2	0	1	2	1	1
**67**	1	0	1	1	1	1	1	1	1
**68**	1	1	2	1	0	1	2	0	0
**69**	1	0	0	0	1	0	0	0	0
**70**	1	2	1	1	1	1	1	1	0
**71**	2	0	0	0	0	0	0	0	1
**72**	0	1	0	1	0	1	1	1	2

#### 4.2.2 RRM model for the selection of the optimum plan

This stage is based on a choice experiment constructed to analyze expert choice of resource allocation plan. The experiment described the allocation plan in terms of three generic attributes—response time, total cost and demand for the black spots. Each attribute was categorized into four levels. The choice set was blocked into 9 choice sets based on the results of the SP model. The experiment, complemented with questions about the importance of these three attributes, was administered via Web-based questionnaires. This study only recruited respondents among a group of people who have studied the emergency management or taken part in related work. The target sample size was 146 respondents. Data collection started on July 10, 2018. The target sample size was achieved on July 19, 2018. 119 valid questionnaires were obtained.

Table 7 reports the frequency distributions of the selected socio-demographic characteristics. It shows that 58.9% of the sample is male, implying 41.1% is female. Age was classified into four categories. The percentages for these categories are respectively 22.3, 36.7, 30.1 and 10.9%. Table 7 also shows that the respondents receive high education in the sample.

**Table 7:** Frequency distribution of socio-demographic characteristics.

Socio-demographic variables		Percentage (%)
**Gender**	Male	58.9
	Female	41.1
**Age**	20–30	22.3
	30–40	36.7
	40–50	30.1
	>50	10.9
**Education**	Undergraduate	17.9
	Master	55.4
	Doctor	26.7
**Whether participated in related decision-making**	Yes	66.1
	No	33.9

In this case, the original regret model and utility-based model were compared with the logarithmic specification to figure out the differences between these models based on different decision rules in Table 8 (See S1 Appendix for the SP model, original regret model and RUM model). For the reason of clear distinction, we use the same software, PandaPython [51, 52], to estimate the parameters.

**Table 8:** Estimation results for different models with the PandaPython.

Parameters	SP-RUM (T-test)	SP-RRM2008 (T-test)	SP-RRM2010 (T-test)
**Response time**	0.186(3.424)***	-0.127(-8.010)***	-0.033(7.240)***
**Total costs**	-0.100(6.785)***	-0.001(-5.019)***	-0.017(-5.170)***
**Demand**	1.020(5.511)***	0.031(1.740)**	0.012(1.770)**
**Null log likelihood**	-261.468	-261.468	-261.468
**Final log likelihood**	-223.8414	-225.471	-213.906
**Rho-square**	0.132	0.126	0.145
**Adjuested rho-square**	0.132	0.126	0.145

Note:

**: robust t-value <0.09

***: robust t-value<0.095.

Table 8 shows the estimation results. The rows show model performance 213indicators and estimates for the three parameters: response time, total costs and demand. The columns show the model types: SP-RUM, SP-RRM2008 and SP-RRM2010.

In terms of model fit, the overall goodness of fit of the RRM2010 model empirically outperforms the standard RUM model and classical RRM model. Log-likelihood is improved by about 10 LL points and 12 LL points as compared to respectively RUM and RRM2008. A statistical test also shows that higher Adj.Rho-square for RRM2010 model means a good overall fitness and a lower Adj.Rho-square for RUM and RRM2008 model illustrates poor fitness.

In a sequel, we turn to the parameter estimates. As expected, the related parameters (i.e. response time, total costs and demand) of the utility-based model and regret-based model are all significant at 90% or 95% level. The positive sign implies that decision makers more highlight the importance of time and demand and the negative sign implies that the total costs are not as important as the time and demand under emergency context. We can apply this parameter estimation to calculate the final alternative results.

Since the SP model is in the mainstream for emergency management, we compare the SP-only model with SP-RRM model and analyze their differences. The results are shown in Table 8. Meanwhile, we focus on empirical studies that report comparisons between RRM and RUM. Therefore, we convert SP model to the utility-based model which added the estimated parameter *β* and observe the differences between SP-RUM and SP-RRM model in Table 8. The overview of the optimal plan ranking based on the different decision rules is shown in Table 9.

**Table 9:** The results of SP-only, SP-RUM and SP-RRM model.

Plan No	T	C	D	SP	SP-RUM	*R*_*T*_	*R*_*C*_	*R*_*D*_	SP-RRM
**1**	3.497	1.436	3.207	2.140	3.779	6.173	6.124	6.224	18.521
**2**	3.365	2.205	0.723	0.293	1.145	6.192	6.183	6.354	18.730
**3**	2.085	1.949	2.793	0.826	3.044	6.385	6.164	6.246	18.795
**4**	3.349	3.231	3.621	4.201	3.997	6.195	6.263	6.203	18.660
**5**	1.988	3.744	3.621	3.352	3.693	6.400	6.303	6.203	18.906
**6**	2.439	4.513	1.965	2.917	2.011	6.332	6.364	6.289	18.984
**7**	2.208	2.462	4.035	2.705	4.283	6.367	6.203	6.181	18.751
**8**	5.336	2.718	3.621	5.675	4.417	5.903	6.223	6.203	18.329
**9**	3.183	4.000	2.793	3.976	0.195	6.220	6.323	6.246	18.789

Note: T, C and D are the dimensional values. The results of RRM is based on the logarithmic specification.

Table 10 reports the optimal plan ranking for the results of SP-only, SP-RUM, and SP-RRM model. As shown in Table 10, Plan2 is the optimal scheme for the SP-only model, but it is the worst one in the SP-RUM and SP-RRM model. As can be seen in Table 9, the attribute of demand extremely compensates the other attributes in terms of SP model. However, the results for the RUM and RRM models could be modified by the estimated parameter *β*. In light of the estimation results, obviously, the attribute of demand is the least consideration among three attributes. The estimated parameters of the SP-RUM and SP-RRM model getting from the experts’ investigations is necessary to assist the decision-maker to balance every important factor. Plan8 is the optimal scheme for the SP-RUM and SP-RRM, which illustrates that SP-RUM and SP-RRM in the emergency context are substantial in final results. Therefore, we choose Plan8 as the final decision.

**Table 10:** The optimal plan ranking for SP-only, SP-RUM and SP-RRM model from small to large.

**SP-only (from small to large)**	2	3	1	6	7	5	9	4	8
**SP-RUM (from large to small)**	8	7	4	1	5	9	3	6	2
**SP-RRM (from small to large)**	8	1	4	2	7	9	3	5	6

## 5. Conclusion and future work

Pre-allocation of emergency supplies can be an effective mechanism for improving response to traffic accidents. We have developed a multi-stage SP-RRM model whose solution provides a pre-allocation strategy for the storage and distribution of emergency supplies under different scenarios. The improved accident frequency method is proposed to identify black spots on the freeway, which increases the accuracy and efficiency for the next work. The emergency supplies pre-allocation approach is a combination of SP model with the constraint of the response time and accident probability and RRM model in the logarithmical specification considering the response time, total cost and demand for black spots.

A case study addressing black spots on the freeways in the three cities of China illustrates both the improved accident frequency and a new SP-RRM model in a practical context. In the case study, after identifying 72 black spots on the freeway in Linyi, Rizhao and Weifang from the historical data in 2014–2016 and calculating their accident probability, we obtained 9 initial plans as our alternatives by SP model and selected the optimum one by RRM model. Moreover, this case study has also allowed experiments to assess the performance of RUM model, RRM2008 model and RRM2010 model. By analyzing the results of the case, we are confident that a multi-stage SP-RRM model can be used as a large-scale resource pre-allocating planning tool.

This paper contributes to analyzing the emergency resources allocation choice based on utility or regret decision rules. The regret model was used to account for the negative psychology, addressing the semi-compensatory issues. In this study, we broke the traditional decision-making method (only SP model) and proposed a RRM model based on the results of the SP models. Meanwhile, in order to improve the defect of the unreasonable weight of important influencing factors in the related literature, we investigated 146 scholars and experts who have studied the emergency management or have taken part in related work. The traditional utility model, regret-based model in the liner-addictive specification and regret-based model in the logarithmic specification are all estimated, respectively. Compared with RUM and RRM2008, an advantage of the RRM2010 was found. It is found that the experts are willing to sacrifice inventory and procurement costs in exchange for safety benefits. However, for the final optimal choice, there is no difference between RUM and RRM models. The findings will provide a new way of thinking from different angles and perspectives for the decision support system and help policy-makers to develop better allocation plans in the emergency context.

Several avenues present themselves as direction for further work. Firstly, one of the problems faced is data available for the casualties of traffic accidents. Due to the confidentiality of accident data, the identification of black spots we proposed didn’t consider the casualties, which impacts the accuracy of the results. In the future study, we will take account into the natural geography locations and the number of casualties on this issue. Secondly, a generalization that treats the multi-commodity flow problem under different weather is certainly worth developing. Although our case highlights the process of allocating one type of supplies on rainy days, our methodology is capable of providing robust preparedness plans for many types of circumstances. Hence, the cases with regard to the pre-allocation of many types of emergency supplies under different scenarios will be proposed to increase the range of potential applicability for the model. Moreover, this paper only illustrates the application of the regret-based model in the field of emergency management, but doesn’t certify RRM models is better than RUM model, or RRM in the linear-additional specification is less than RRM in logarithmical specification, because we are based on a single dataset and based on one scenario. In the future, we will compare with different decision rules based on several databases theoretically and empirically. In addition, the limitations of comparison is our method is a time-consuming task. It relies on the preference and experiences of the experts and often ignores the actual conditions of the specific areas. In future work, we may improve our method and be better applied in the engineering project.

## Supporting information

S1 Fig The attribute layer of regret function based on *β*_*m*_ and (*x*_*jm*_−*x*_*im*_). (PDF)

S2 Fig Sketch of cut section technology. (PDF)

S3 Fig The proportion of accident on every freeway. (PDF)

S1 Appendix (DOCX)

S1 File Part of the accident data. (XLSX)

S2 File Part of the result of questionnaire. (XLSX)
