# Supplementary figures and images for: A multi-stage emergency supplies pre-allocation approach for freeway black spots: A Chinese case study
Source: PLoS One. 2020 Oct 8;15(10):e0240372. doi: 10.1371/journal.pone.0240372 (PMC7544114; doi:10.1371/journal.pone.0240372)

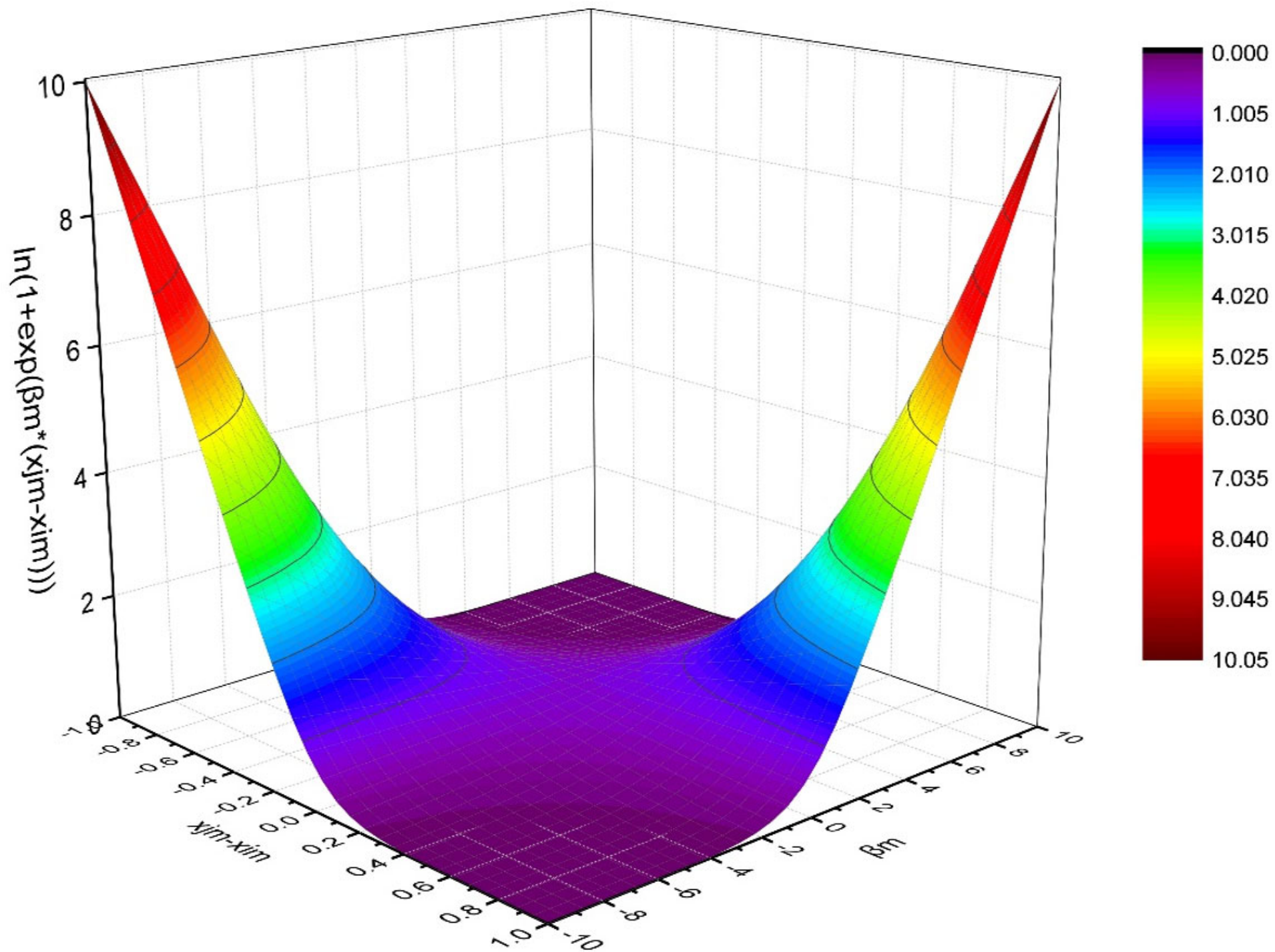

Supplement: S1 Fig — (PDF) [file pone.0240372.s001.pdf]

## Condition 1:

The number of  
accident

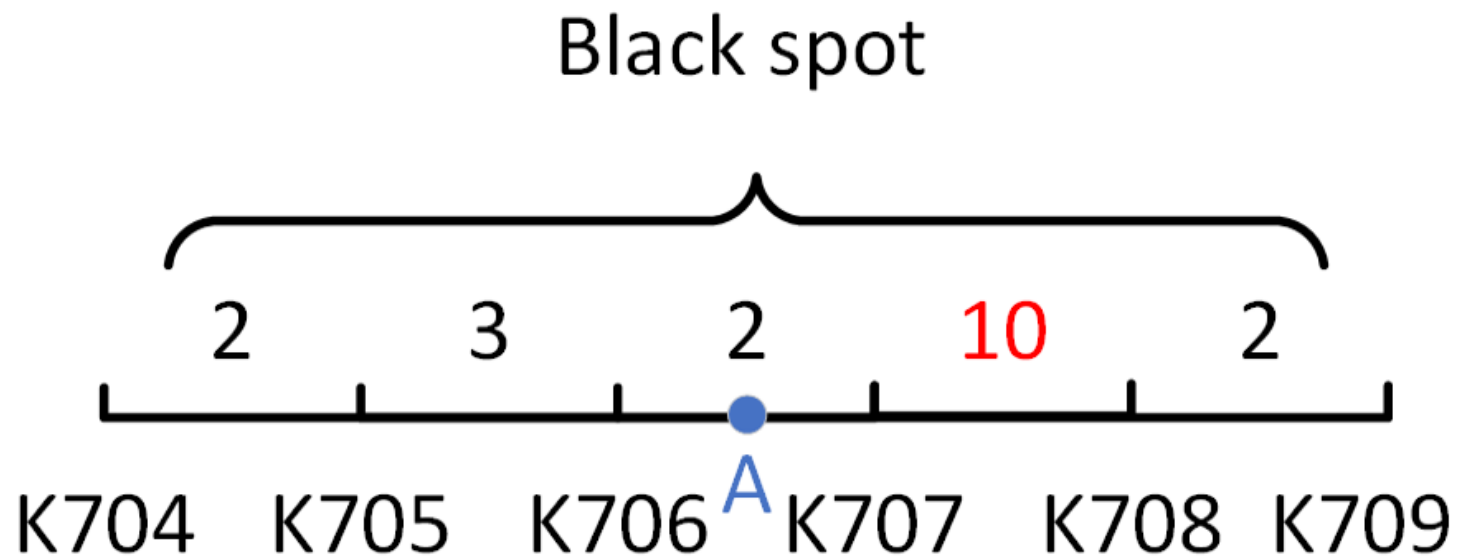

## Condition 2:

The number of  
accident

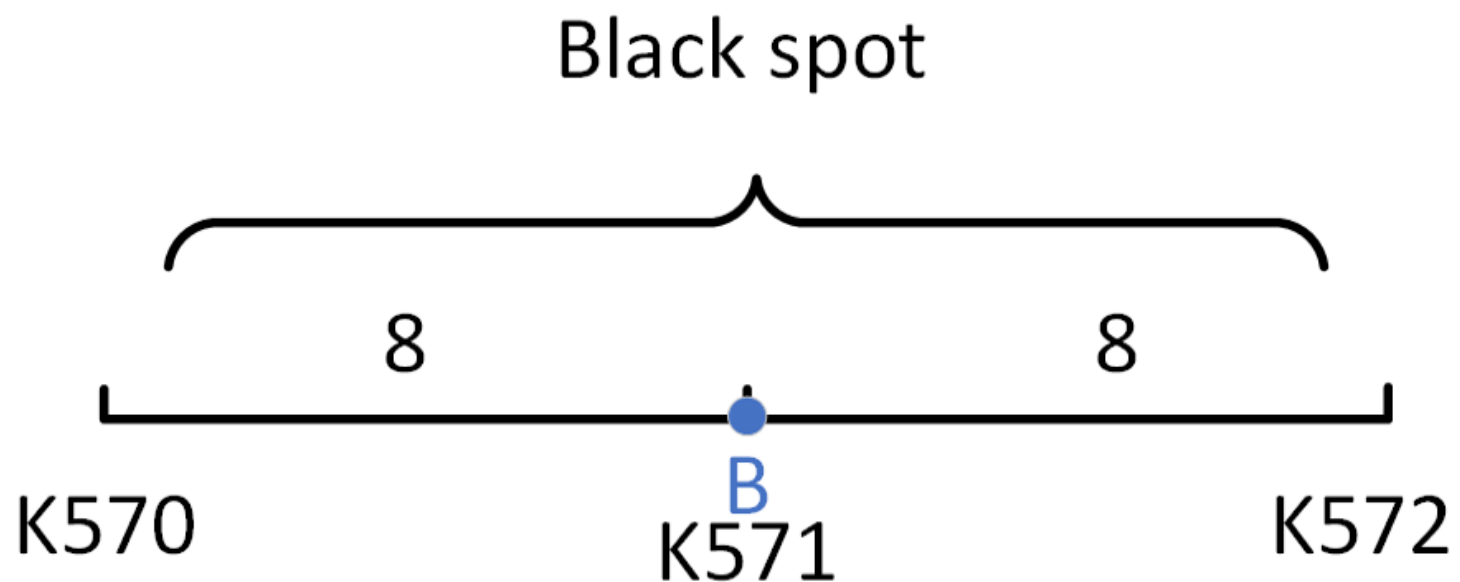

Supplement: S2 Fig — (PDF) [file pone.0240372.s002.pdf]

# PERCENTAGE

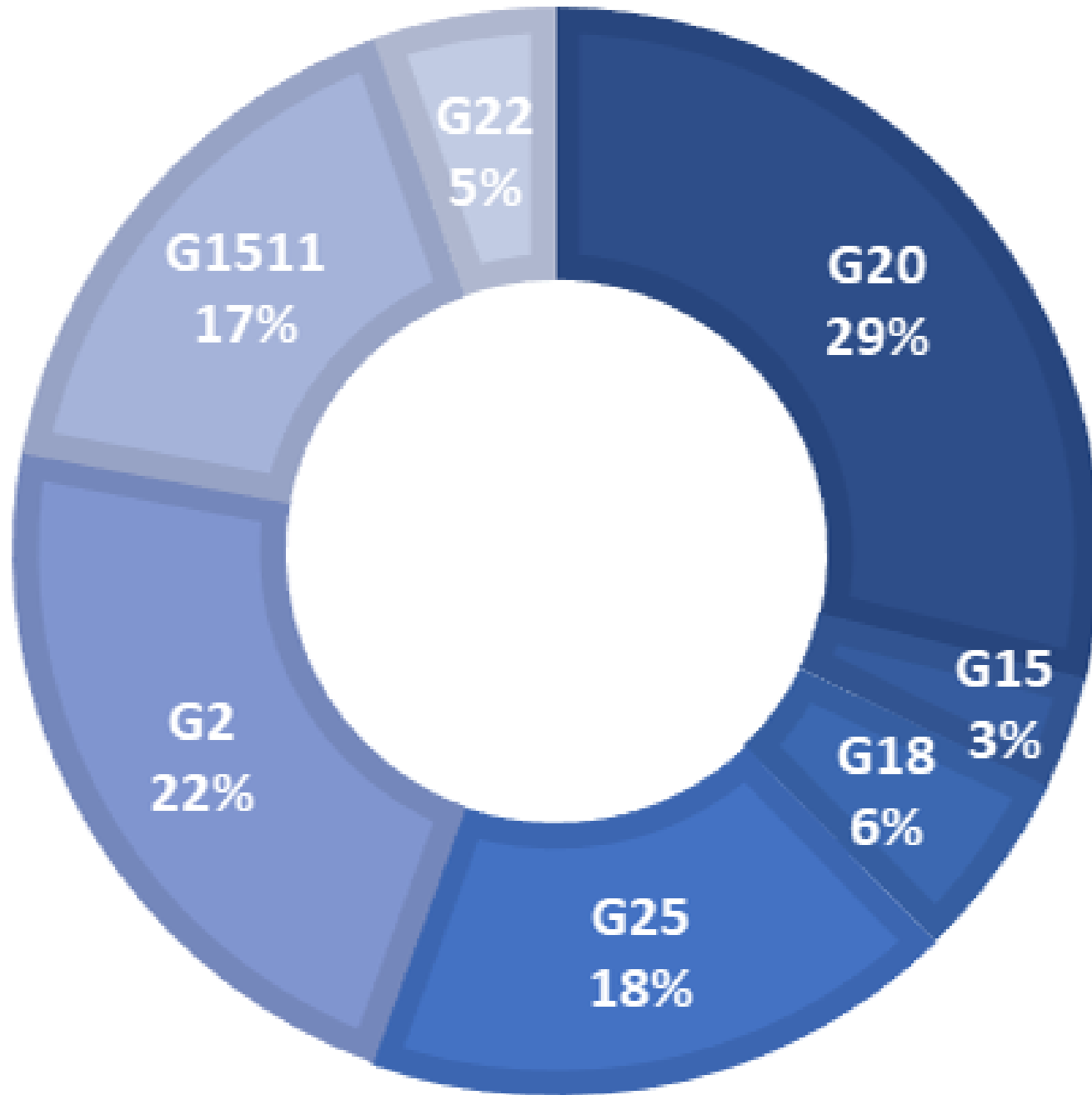

Supplement: S3 Fig — (PDF) [file pone.0240372.s003.pdf]
