# Supplementary material for: A multi-stage emergency supplies pre-allocation approach for freeway black spots: A Chinese case study
Source: PLoS One. 2020 Oct 8;15(10):e0240372. doi: 10.1371/journal.pone.0240372 (PMC7544114; doi:10.1371/journal.pone.0240372)
Supplement: S1 Appendix — (DOCX) [file pone.0240372.s004.docx]

**Appendix 1: The original regret-model specification, the utility-model specification and the SP-only model**

This RRM-specification illustrated that deterministic regret equals the maximum of all alternatives’ regrets that are judged against the best alternative for each attribute for each attribute separately. Mathematical expressions combining with this paper are as follows:

$$\min R_{n}(\xi)$$

$$R_{1}\text{=max}\left\{R_{12},R_{13},\cdots,R_{1m}\right\}$$

$$R_{2}\text{=max}\left\{R_{21},R_{23},\cdots,R_{2m}\right\}$$

⋮

$$R_{m}\text{=max}\left\{R_{m1},R_{m2},\cdots,R_{m(m-1)}\right\}$$

$$T_{n}=\sum_{i=1}^{n_{i}} \sum_{j=1}^{n_{j}} t_{ij}x_{ij}$$

$$C_{n}=\sum_{i=1}^{n_{i}} \sum_{j=1}^{n_{j}} \left(C_{s}+C_{b}\right)G_{i}x_{ij}$$

$$W_{n}=\sum_{j=1}^{n_{j}} (d_{j}-x_{j})$$

Subject to:

$$\sum_{j\in J} x_{ij}(\xi)\leq a_{i},\forall i\in I$$

$$\sum_{i\in I} \sum_{j\in J} x_{ij}(\xi)\leq A$$

$$\sum_{j\in J} x_{ij}(\xi)\geq r,\forall i\in I$$

$$\sum_{i\in I} x_{ij}(\xi)\leq d_{j},\forall j\in J$$

$$\sum_{i\in I} \sum_{j\in J} \left(C_{s}+C_{b}\right)G_{i}x_{ij}(\xi)\leq B$$

$x_{ij}(\xi)\geq0$, and integer $\forall i\in I$

Given iid Extreme Value Type I errors added to deterministic regret, the choice probability is shown as follows:

$$P_{i}=\frac{\exp(-R_{i})}{\sum_{j=1...J} \exp(-R_{j})}$$

For the utility-based model, the objective function associated with the final stage of emergency resources allocation is written as:

$$\max U(\xi)$$

$$U_{n}(\xi)=\beta_{T}\cdot T_{n}+\beta_{C}\cdot C_{n}+\beta_{D}\cdot D_{n}+\varepsilon$$

For the SP-only model, the objective function compared with the SP-RRM model is expressed as:

$$\min\sum_{i\in I} \sum_{j\in J} \theta_{ij}(\xi)p_{j}(\xi)[t_{ij}(\xi)x_{ij}(\xi)+\left(C_{s}+C_{b}\right)G_{i}x_{ij}(\xi)+\left(d_{j}-x_{ij}(\xi)\right)]$$
